# Cubital Tunnel Syndrome Temporally after COVID-19 Vaccination

**DOI:** 10.3390/tropicalmed7040062

**Published:** 2022-04-16

**Authors:** Luca Roncati, Davide Gravina, Caterina Marra, Norman Della Rosa, Roberto Adani

**Affiliations:** 1 Institute of Pathology, Department of Surgery, Medicine, Dentistry and Morphological Sciences with Interest in Transplantation, Oncology and Regenerative Medicine, University of Modena and Reggio Emilia, 41125 Modena, Italy; 2 Unit of Orthopedics and Traumatology, Department of Musculoskeletal System, University Hospital of Modena, 41125 Modena, Italy; davide.gravina@unimore.it; 3 Unit of Plastic and Reconstructive Surgery, Department of General Surgery and Surgical Specialties, University Hospital of Modena, 41125 Modena, Italy; caterina.marra@unimore.it; 4 Unit of Hand Surgery and Microsurgery, Department of Musculoskeletal System, University Hospital of Modena, 41125 Modena, Italy; dellarosa.norman@aou.mo.it (N.D.R.); adani.roberto@aou.mo.it (R.A.)

**Keywords:** coronavirus disease 2019 (COVID-19), severe acute respiratory syndrome coronavirus 2 (SARS-CoV-2), vaccines, modified messenger ribonucleic acid (modRNA), Spikevax^®^, modRNA COVID-19 vaccine, cubital tunnel syndrome, ulnar neuropathy, Alcian Blue stain, histochemistry

## Abstract

Coronavirus disease 2019 (COVID-19) is the most dramatic pandemic of the new millennium. To counter it, specific vaccines have been launched in record time under emergency use authorization or conditional marketing authorization and have been subjected to additional monitoring. The European Medicines Agency recommend reporting any suspected adverse reactions during this additional monitoring phase. For the first time in the available medical literature, we report a left cubital tunnel syndrome in a 28-year-old right-handed healthy male after seven days from the first dose of Spikevax^®^ (formerly Moderna COVID-19 Vaccine). Histochemistry for Alcian Blue performed on the tissue harvested from the cubital site reveals myxoid degeneration of the small nerve collaterals, a clear sign of nerve injury. It still remains unclear why the syndrome occurs in a localized and not generalized form to all osteofibrous tunnels. Today, modified messenger ribonucleic acid vaccines as Spikevax^®^ represent an avantgarde technological platform with a lot of potential, but one which needs careful monitoring in order to identify in advance those patients who may experience adverse events after their administration.

## 1. Introduction

Coronavirus disease 2019 (COVID-19) is the most dramatic pandemic of the new millennium. To counter it, specific vaccines have been launched in record time under emergency use authorization or conditional marketing authorization [1]. For the first time in the history of medicine, millions of people have been so inoculated with new generation vaccines, namely modified messenger ribonucleic acid (modRNA) vaccines, in the course of an unprecedented vaccine campaign [2].

Among these vaccines, there is Spikevax^®^ (formerly Moderna COVID-19 Vaccine), a single-stranded 5′-capped modRNA produced using a cell-free in vitro transcription from the corresponding deoxyribonucleic acid templates, encoding the spike protein of the severe acute respiratory syndrome coronavirus 2 (SARS-CoV-2), the well-known etiological agent of COVID-19 [3].

A 0.5 mL dose of Spikevax^®^ contains 100 µg of modRNA embedded in heptadecan-9-yl 8-{(2-hydroxyethyl)[6-oxo-6-(undecyloxy)hexyl]amino}octanoate (SM-102) lipid nanoparticles, plus 1,2-dimyristoyl-rac-glycero-3-methoxypolyethylene glycol-2000 (PEG2000 DMG), 1,2-distearoyl-sn-glycero-3-phosphocholine (DSPC), acetic acid, cholesterol, sodium acetate, sucrose, trihydrate, trometamol, trometamol hydrochloride and water for injections [3].

As for all medicinal products, careful pharmacovigilance in the short, medium and long term is required. Moreover, national and supranational drug regulatory agencies, such as the European Medicines Agency, recommend reporting any suspected adverse reaction during the phase of additional monitoring [3]. From this tracking activity, COVID-19 vaccination has emerged as one possible rare trigger of Guillain–Barré syndrome, the well-known acute peripheral demyelinating polyneuropathy on an autoimmune basis, resulting in rapid-onset generalized muscle weakness. In about 15% of patients, the involvement of breathing muscles occurs with the need for mechanical ventilation [4,5,6,7,8].

## 2. Case Report

A 28-year-old right-handed Caucasian male intolerant to salicylates and local anesthetics devoid of relevant pathologies went to the orthopedic emergency room for the sudden appearance of paresthesia to the left upper limb accompanied by slight “hand blessing” attitude. Seven days earlier, he had received the first dose of Spikevax^®^ on the same side.

After a careful examination, no sign of central neurological impairment was found, and the clinical picture appeared consistent with a peripheral ulnar neuropathy characterized by involvement of the ulnar metamerum of the left hand and of the intrinsic musculature at the metacarpal bone III. In light of this, the patient was submitted to electromyography for ulnar and median nerve, which showed a mononeuropathy of the left ulnar nerve at the elbow of medium degree (cubital tunnel syndrome). More in detail, the sensory action potential of the dorsal branch of the left ulnar nerve was absent, and a partial block of conduction of the motor portion of the left ulnar nerve in the transition to the elbow was highlighted. In addition, electrical silence was detected at rest on stimulation of the short abductor muscles of the thumb and the left first dorsal interosseus. By virtue of all this, a grade of II according to the McGowan scale was assigned to the patient (Table 1) [9].

Before decompression surgery, an anti-nucleocapsid SARS-CoV-2 γ-immunoglobulins (IgG) test and an anti-spike receptor binding domain (RBD) SARS-CoV-2 IgG test were performed by chemiluminescent microparticle immunoassay (CMIA). The former resulted negative (0.220 UA/mL), while the latter was positive (5816.30 UA/mL).

**Figure 1:**
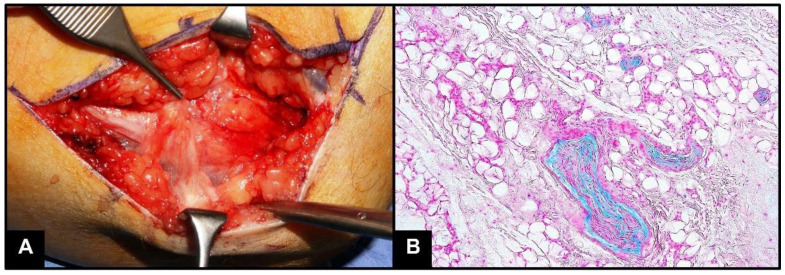
(**A**). Macroscopic intraoperative photography showing ulnar nerve entrapment at the left elbow (cubital tunnel syndrome). (**B**). On microscopy, histochemistry for Alcian Blue reveals in light blue myxoid degeneration of the small nerve collaterals (20× objective).

During surgery, entrapment was confirmed (Figure 1A), and the tissue around the compression site was harvested and sent for histological examination. Histochemistry for Alcian Blue revealed myxoid degeneration of the small nerve collaterals (Figure 1B), a clear sign of nerve injury.

Subsequently, the patient opted not to receive the second dose of Spikevax^®^, thus not completing the primary vaccination course, nor to receive a booster dose.

## 3. Discussion

Spikevax^®^ is indicated for active immunization to prevent COVID-19 in individuals 12 years of age and older. It is administered as primary course of two doses at a time distance of 28 days from each other [3]. A booster dose (0.25 mL) may be administered intramuscularly at least 5–6 months after the second dose in individuals 18 years of age and older [3].

Among the reported adverse reaction there are: arthralgia (≥1/10) [3], chills (≥1/10) [3], fatigue (≥1/10) [3], myalgia (≥1/10) [3], nausea/vomiting (≥1/10) [3], headache (≥1/10) [3], lymphadenopathy (≥1/10) [3], pyrexia (≥1/10) [3], injection site pain or swelling (≥1/10) [3], injection site erythema or delayed reaction (≥1/100 to <1/10) [3], diarrhea (≥1/100 to <1/10) [3], rash (≥1/100 to <1/10) [3], dizziness (≥1/1000 to <1/100) [3], injection site pruritus (≥1/1000 to <1/100) [3], acute peripheral facial paralysis (≥1/10,000 to <1/1000) [3], facial swelling (≥1/10,000 to <1/1000) [3], myocarditis (<1/10,000) [3], pericarditis (<1/10,000) [3], erythema multiforme or nodosum (frequency not known) [3,10], pityriasis rosea (frequency not known) [11,12], pemphigus vulgaris (frequency not known) [13], bullous pemphigoid (frequency not known) [14,15,16], acantholytic dyskeratosis (frequency not known) [17], vitiligo (frequency not known) [18], livedo reticularis (frequency not known) [19], herpes reactivation (frequency not known) [20,21,22], multiple sclerosis (frequency not known) [23], Guillain-Barré syndrome (frequency not known) [24,25,26,27], Churg-Strauss syndrome [28], Löfgren syndrome (frequency not known) [29], Sweet syndrome (frequency not known) [30], Takotsubo syndrome (frequency not known) [31], Moyamoya disease (frequency not known) [32], capillary leak syndrome (frequency not known) [33,34], vasculitis (frequency not known) [35,36], thrombosis (frequency not known) [37,38,39], thrombotic thrombocytopenia (frequency not known) [40,41,42,43,44,45,46,47], pulmonary embolism (frequency not known) [48,49], aplastic or autoimmune hemolytic anemia (frequency not known) [50,51,52], diffuse alveolar hemorrhage (frequency not known) [53], auto-immune hepatitis (frequency not known) [54,55,56], encephalopathy (frequency not known) [57,58], acute transverse myelitis (frequency not known) [59], subacute thyroiditis (frequency not known) [60,61], retinal detachment (frequency not known) [62,63,64], anterior uveitis (frequency not known) [65], orbital inflammation (frequency not known) [66], sensorineural hearing loss (frequency not known) [67], α-immunoglobulins (IgA) nephropathy (frequency not known) [36,68], anti-neutrophil cytoplasmic antibodies (ANCA) glomerulonephritis (frequency not known) [69], urticaria (frequency not known) [70,71], anaphylaxis (frequency not known) [72,73,74], and a whole series of autoimmune exacerbations among which systemic lupus erythematosus, psoriasis, myasthenia gravis, polymyalgia rheumatica, and dermatomyositis [75,76,77,78,79,80].

Previously, our research group has described two cases of concomitant cubital and carpal syndrome after severe COVID-19 in the non-dominant limb of as many middle-aged male patients admitted to intensive care [81]. Similarly, Terhoeve and colleagues have reported three cases of ulnar nerve palsy as sequelae of severe COVID-19 [82].

Today, anti-nucleocapsid SARS-CoV-2 IgG CMIA is the serological test of choice to evaluate long-term SARS-CoV-2 infection [83], while anti-spike RBD SARS-CoV-2 IgG CMIA finds application to ascertain the immune response after vaccination [84]. The antibodies profile of our patient testifies that he underwent COVID-19 vaccination, but he didn’t contract the virus. Therefore, the question arises concerning whether naturally or vaccine-induced anti-spike antibodies may play a role in the pathogenesis of these rare complications, both characterized by myxoid (alias mucoid or mucinous) nerve degeneration on histology. With the few data in our possession (the main limitation of our study), it appears premature to provide a conclusive answer.

When compared to Guillain-Barré syndrome, which begins in the hands and feet ascending to the arms, legs and upper body within hours or days, it still remains unclear why these events occur in a localized and not generalized form to all osteofibrous tunnels.

## 4. Conclusions

Our work attempts to describe in depth a new neurological event to keep in mind during COVID-19 vaccination schedule. It allows to add a piece in the complex spectrum of post-vaccinal neurological events, that vary from paresthesia to more serious conditions such as Guillain-Barré syndrome.

Tested on humans starting from 2005 at first to fight cancer and then from 2013 against infectious agents, modRNA vaccines represent today an avantgarde technological platform with a lot of potential, but one which needs careful monitoring in order to identify in advance those subjects who may experience more or less serious adverse events after their administration.

## Figures and Tables

**Table 1:** The McGowan scale for the grading of cubital tunnel syndrome: grade I corresponds to a minimal lesion, grade II to an intermediate lesion, and grade III to a severe lesion.

**GRADE I**	**PURELY SUBJECTIVE SYMPTOMS**
**GRADE II**	**MUSCLE WEAKNESS AND/OR OBJECTIVE SENSORY SIGNS**
**GRADE III**	**SIGNIFICANT SENSORY AND MOTOR DEFICITS WITH NOTICEABLE ATROPHY OF INTRINSIC MUSCLES**

## Data Availability

The data presented in this study are available on request from the corresponding author.
